# Transcriptomic exploration yields novel perspectives on the regulatory network underlying trichome initiation in *Gossypium arboreum* hypocotyl

**DOI:** 10.3389/fpls.2025.1604186

**Published:** 2025-07-02

**Authors:** Yuxing Xie, Luying Yang, Zewei Zhao, Mingquan Ding, Yuefen Cao, Xin Hu, Junkang Rong

**Affiliations:** College of Advanced Agricultural Sciences, Zhejiang A&F University, Linan, Hangzhou, Zhejiang, China

**Keywords:** Gossypium arboreum, trichome initiation development, transcriptome analysis, WGCNA, machine learning

## Abstract

Trichomes play a crucial role in plant stress tolerance and serve as an excellent model for studying epidermal cell differentiation. To elucidate the molecular mechanisms underlying trichome development in cotton stems, we investigated two Gossypium arboreum mutants that exhibit abnormal trichome patterns during hypocotyl growth. Based on morphological characteristics, we classified four developmental stages: preinitiation, initiation, elongation, and maturation. Comparative transcriptome profiling of epidermal cells across these stages identified differentially expressed genes (DEGs) through maSigPro analysis, which revealed that these DEGs were primarily associated with pathways involved in cell wall metabolism. Additionally, integrated weighted gene co-expression network analysis (WGCNA) and Cytoscape analyses identified 20 core regulatory genes from a total of 59 candidates linked to epidermal development. Utilizing three machine learning algorithms (SVM-RFE, Boruta, and LASSO), we consistently prioritized five key regulators: *Ga02G1392 (TBR), Ga03G0474 (OMR1), Ga12G2860 (ACO1), Ga11G2117 (BBX19), and Ga12G2864 (CUE).* RT-qPCR validation confirmed their stage-specific expression patterns, which were consistent with the RNA-Seq data. Our study establishes a comprehensive framework for research on cotton trichomes and identifies critical genetic components governing epidermal hair development, thereby providing new insights for the molecular breeding of stress-resistant cotton varieties.

## 1 Introduction

Trichomes, which are surface hairs that arise from the protrusion of plant epidermal cells, can be functionally categorized into glandular and non-glandular types. Glandular hairs form a chemical barrier by secreting secondary metabolites, while non-glandular hairs enhance plant adaptability to extreme temperatures and biological stress through physical barriers (Ding et al., 2020; Feng et al., 2019). Investigating the molecular regulatory mechanisms underlying the differentiation and development of plant epidermal cells represents a critical area of biological research, with the establishment of an optimal research model serving as a foundational strategy for unraveling these complex biological processes. Since the 1990s, epidermal hairs of *Arabidopsis thaliana* have emerged as a quintessential model system for studying plant cell differentiation and morphogenesis, owing to their unique morphological features and well-defined developmental pathways. Significant progress has been made in understanding aspects such as cell fate determination, cell cycle regulation, cell polarity establishment, and cell shape modulation (Hulskamp, 2004).

In *Arabidopsis*, the morphological changes of epidermal hair cells can be categorized into three stages based on their orderly spatial distribution: the initiation of nuclear replication, the establishment of the polarity axis, and the development of branching morphology (Bouyer et al., 2001). By leveraging the established trichome system, researchers identified that the GLABRA1 (GL1) - GLABRA3 (GL3) - TRANSPARENT TESTA GLABRA1 (TTG1) complex forms a feedback regulatory loop by activating downstream R3 MYB inhibitory factors, which precisely regulates the spatiotemporal specificity of epidermal hair initiation (Schwab et al., 2000). Simultaneously, the C2H2 zinc finger protein GIS plays a crucial role in determining epidermal hair fate by modulating the activity of the MYB-bHLH-WDR complex, thereby unveiling a novel mechanism of synergistic regulation that integrates epigenetic modifications and transcriptional networks (Zhang et al., 2018).

In contrast to the single-cell model, the morphological development of multicellular epidermal hairs, such as those found in cucumber and tomato, entails more intricate processes of cell division and differentiation. In tomato (*Lycopersicon esculentum*), the *Hairless-2* (*HL-2*) gene interacts with the HD-zip IV transcription factor *SIHDZIV8* to modulate the morphology of multicellular epidermal hairs by regulating actin cytoskeleton remodeling and cellulose synthesis (Xie et al., 2020). Similarly, in cucumber (*Cucumis sativus*), the molecular basis of functional differentiation in epidermal hairs has been elucidated; specifically, *CSHOMEOBOX3* (*CsHOX3*) plays a role in the elongation of non-glandular hairs, while *CsbHLH1* is implicated in the formation of apical secretory structures within glandular hairs (Dong et al., 2022). Moreover, the receptor-like kinase *CsTM* (*CSA1G056960*) has been shown to establish a direct connection between the morphological development of the surface coat and aphid resistance by integrating mechanical signals and redox homeostasis (Yang et al., 2025).

The single-cell development model established in cotton (Qin et al., 2022) provides a comprehensive analysis of the dynamic regulatory network influencing fiber cell development from primordium initiation (2–5 days post-anthesis, DPA) and polar elongation (3–20 DPA) to secondary wall thickening (16–40 DPA) (Feng et al., 2018). The MBW complex plays a crucial role in this process, with various transcription factors, including the R2R3-type MYB transcription factors *GhMYB109* (Wang L, et al., 2021), *GhMYB25* (Wu et al., 2006), and *GhMYB25-like* (Walford et al., 2011; Wan et al., 2016), as well as the HD-Zip family transcription factors *GhHD1* (Walford et al., 2012) and *GhHOX3* (Shan et al., 2014), positively regulating cotton fiber initiation. Moreover, the NAC-MYB-CESAs module is instrumental in secondary wall synthesis by modulating cellulose synthase activity (Taylor-Teeples et al., 2015; Cao et al., 2020). Additionally, plant hormones have a significant impact on the development of surface hairs, as demonstrated by the overexpression of *GhPIN3*, which enhances fiber-specific auxin (AUX) accumulation (Ma et al., 2019; Zhang et al., 2017b), thereby facilitating fiber initiation and development (Li et al., 2007; Zhang et al., 2017a). Flavanone 3-hydroxylase (F3H)-related products (Naoumkina et al., 2015; Tan et al., 2013) and other flavonoids also play critical roles in fiber development by influencing the distribution, activity, and content of auxin carriers, as well as altering the levels of reactive oxygen species (ROS) and the activity of associated proteins. This intricate interplay ultimately affects the progression of fiber development (Edward, 2013; Silva-Navas et al., 2016; Wang et al., 2015). Collectively, these studies highlight that the formation of unicellular trichomes differs from that of multicellular trichomes and is governed by a diverse array of genes.

Numerous studies have demonstrated that RNA transcriptional regulation plays a critical role in plant growth and development. RNA sequencing technology has become widely utilized in crop research to analyze gene expression patterns and elucidate the molecular mechanisms underlying various traits (Aleem et al., 2021; Li et al., 2017; Shankar et al., 2016). While research on the initiation and development of cotton trichomes has primarily concentrated on cotton fibers (Qin et al., 2022), significant morphological differences exist between cotton stem hairs and cotton fibers, suggesting that the developmental mechanisms of trichomes may differ (Hu et al., 2019). Although some studies have reported quantitative trait locus (QTL) mapping and have cloned several key genes that regulate cotton epidermal hairs (Ding et al., 2015; Wan et al., 2007), the underlying molecular mechanisms remain poorly understood.

In this study, we selected two mutants of *Gossypium arboreum*: SMA-4, a *GaHD1* loss-of-function mutant, and DPL972, a *GaGIR1* overexpression mutant, as our research subjects. We developed a system for investigating epidermal hair initiation and development at the cellular level during hypocotyl growth, which, in combination with transcriptome analysis of epidermal cells, led us to identify five key genes. We examined the effects of these genes on trichome growth and development, as well as the regulatory networks involved in epidermal hair initiation and development. Our findings provide a theoretical foundation for a deeper understanding of the molecular mechanisms that govern the complex processes underlying cotton epidermal hair initiation and development. Moreover, our research offers valuable insights for future investigations into single-cell trichome development.

## 2 Materials and methods

### 2.1 Plant materials

In this study, we utilized the Asian cotton varieties SMA-4, DPL972, and A2–47 as plant materials. SMA-4 is a *GaHD1* mutant that exhibits a loss of function (Ding et al., 2020), resulting in the absence of epidermal hairs on the stems, leaves, and fibers. DPL972 is a mutant characterized by high expression of *GaGIR1* (Wang X, et al., 2021), leading to short trichomes on the stem and long fibers on the seed, while lacking long trichomes and short fibers. In contrast, A2–47 is an Asian cotton variety that possesses both normal long and short trichomes on the stem, as well as long and short fibers on the seed.

### 2.2 Technical process

In this study, we observed the epidermal hairs on the hypocotyls of Asian cotton and analyzed the associated molecular mechanisms using RNA-seq and RT-PCR techniques, as outlined in (**Supplementary Figure S1**). Following phenotypic observations, we conducted principal component analysis (PCA) and differential expression analysis of genes (DEGs). MaSigPro time series analysis was utilized to identify differentially expressed genes among materials at various time points, while Weighted Gene Co-expression Network Analysis (WGCNA) was employed to identify key gene modules. The results from MaSigPro time series analysis were integrated with other relevant data using a Venn diagram. For feature selection, we applied Least Absolute Shrinkage and Selection Operator (LASSO) regression analysis, Support Vector Machine Recursive Feature Elimination (SVM-RFE) analysis, and Boruta analysis. Through this comprehensive series of analytical steps, we identified the DEGs associated with the growth and development of hypocotyl trichomes.

### 2.3 Scanning electron microscopy observation

**Figure 1:**
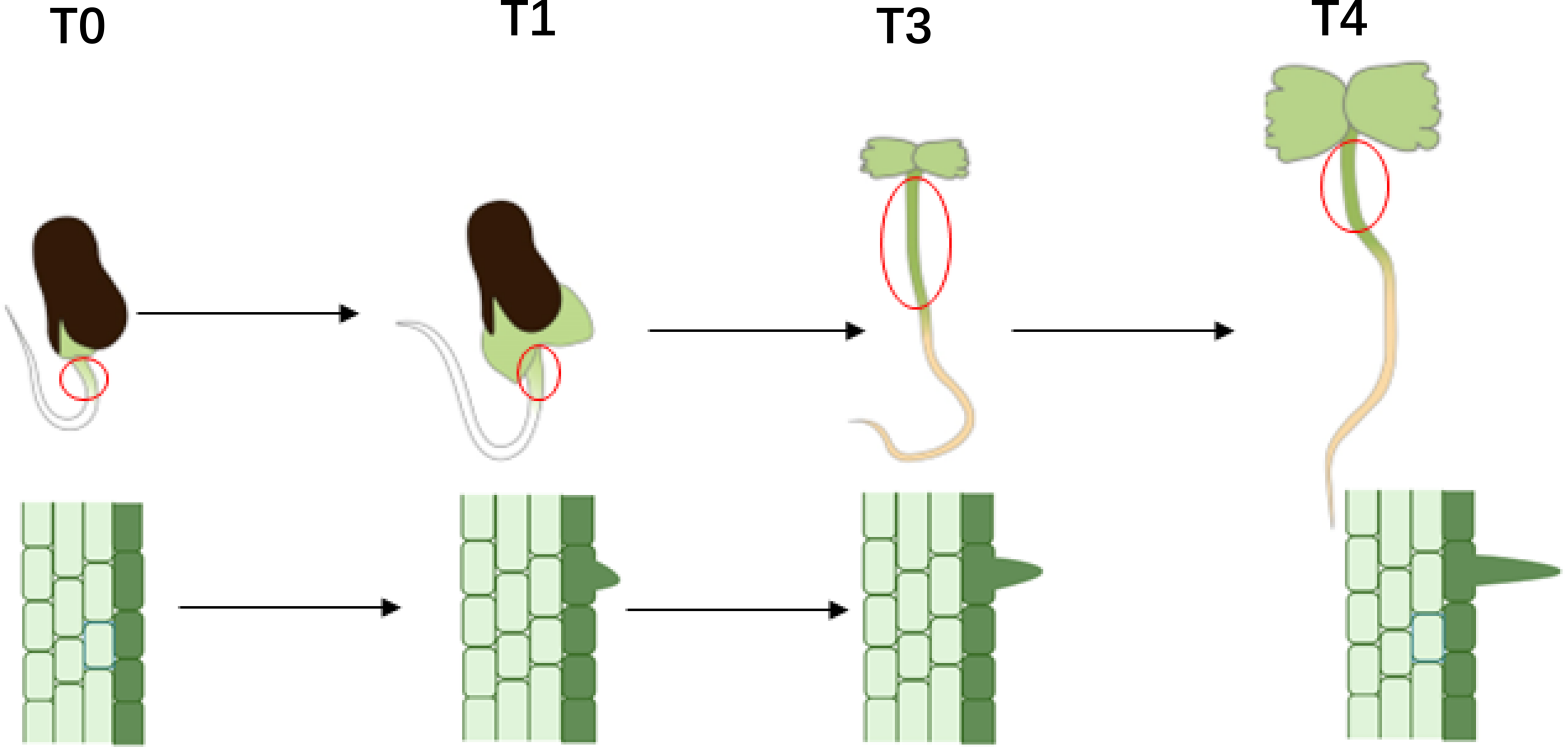
Trichome initiation and development pattern on the hypocotyl of *Gossypium arboreum*.

To investigate the initial development of hypocotyl trichomes, cotton seeds were soaked in water for 8 to 12 hours and then placed on germination paper. After the radicle had emerged, samples were collected at 24, 60, 72, and 104 hours, subsequently referred to as T0, T1, T3, and T4. The seeds were examined under a stereomicroscope to document the early trichome development. The epidermal cells of the hypocotyl at these four developmental stages were carefully removed with tweezers (**Figure 1**) and immediately placed in liquid nitrogen to prevent RNA degradation. Additionally, the epidermal cells corresponding to these four stages were prepared for microscopy, and the morphology of the trichomes was analyzed using a scanning electron microscope (HITACHI S-530) (Hu et al., 2018).

### 2.4 RNA - seq data analysis

At T0, T1, T3, and T4, the epidermis of the growing portion of the epicuticular hairs on the hypocotyl was removed, RNA was extracted, and both mRNA and sRNA libraries were constructed following the guidelines provided in the Illumina Transcription Library Manual. Each sample consisted of three biological replicates, resulting in a total of 36 libraries. The libraries were sequenced using the Illumina Novaseq platform, employing double-ended sequencing technology.

Use Cutadapt and FASTQ (Chen et al., 2018) software for quality control of raw data. Hisat2 software (version 2.2.1) (Kim et al., 2015) was used to map clean reads to the reference gene of Asian cotton (G. arboreum _ CRI_ A2, version 1.0). SAMTOOLS (v.0.1.19; Danecek et al., 2021) was used to filter sequenced reads with mapping quality < 25. The rest used R language (version 4.2.2) to calculate the TPM value of the sample (**Supplementary Table S1**). Using DESeq2 (version 1.26.0) (Love et al., 2014) and |log2 (fold change)| ≥ 1 and P-adjusted < 0.05 as screening conditions, DEGs were identified.

### 2.5 WGCNA

Co-expression networks were constructed using WGCNA (version 1.70) (Langfelder and Horvath, 2008) for all genes in 36 samples. The power was 8, minModuleSize was 30, mergeCutHeight was 0.25, and the rest of the modules were constructed using the default settings of the automatic network construction function blockwiseModules. Among the identified modules, the gray modules representing genes with classification failure are considered invalid. Modules with |cox| > 0.3 and P value < 0.05 were highly associated with the phenotype. Finally, the module genes were intersected with the differential analysis taken to construct the interaction network of intersected genes and GaHD1, GaGIR1 using Cytoscape software (version 3.9.0) (Otasek et al., 2019).

### 2.6 Machine learning

SVM-RFE analysis, Boruta’s algorithm and LASSO regression analysis are statistical methods for feature selection and regression. See reference for a detailed description (Desai et al., 2008; Friedman et al., 2010; Kursa and Rudnicki, 2010). The intersecting genes of WGCNA and temporal analysis were algorithmically analyzed by constructing a model featuring velvet phenotypes at different stages of the hypocotyl to achieve automated feature selection to obtain more precise genes related to the initiation of velvet development in cotton.

### 2.7 Quantitative real-time PCR verification of candidate genes

The hypocotyl epidermal cells of A2-47, SMA-4, and DPL972 at T0, T1, T3, and T4 during the initial development of hypocotyls were taken as samples. The TB Green^®^ Premix Ex TaqTM II (Tli RNase Plus) kit (Vazyme, R223-01) was utilized to transcribe 1 μg of RNA from each sample into complementary DNA (cDNA). Specific primers (**Supplementary Table S2**) were designed utilizing the https://biodb.swu.edu.cn/qprimerdb/website. The amplification program consisted of an initial denaturation step at 95°C for 30 seconds, followed by 39 cycles of denaturation at 95°C for 5 seconds and annealing at 60°C for 30 seconds. The relative expression levels of the genes were determined using the 2^-ΔΔCt^ method, as described by (Livak and Schmittgen, 2001).

## 3 Results

### 3.1 Initial development of epidermal hairs during hypocotyl elongation in *Gossypium arboreum L.*

**Figure 2:**
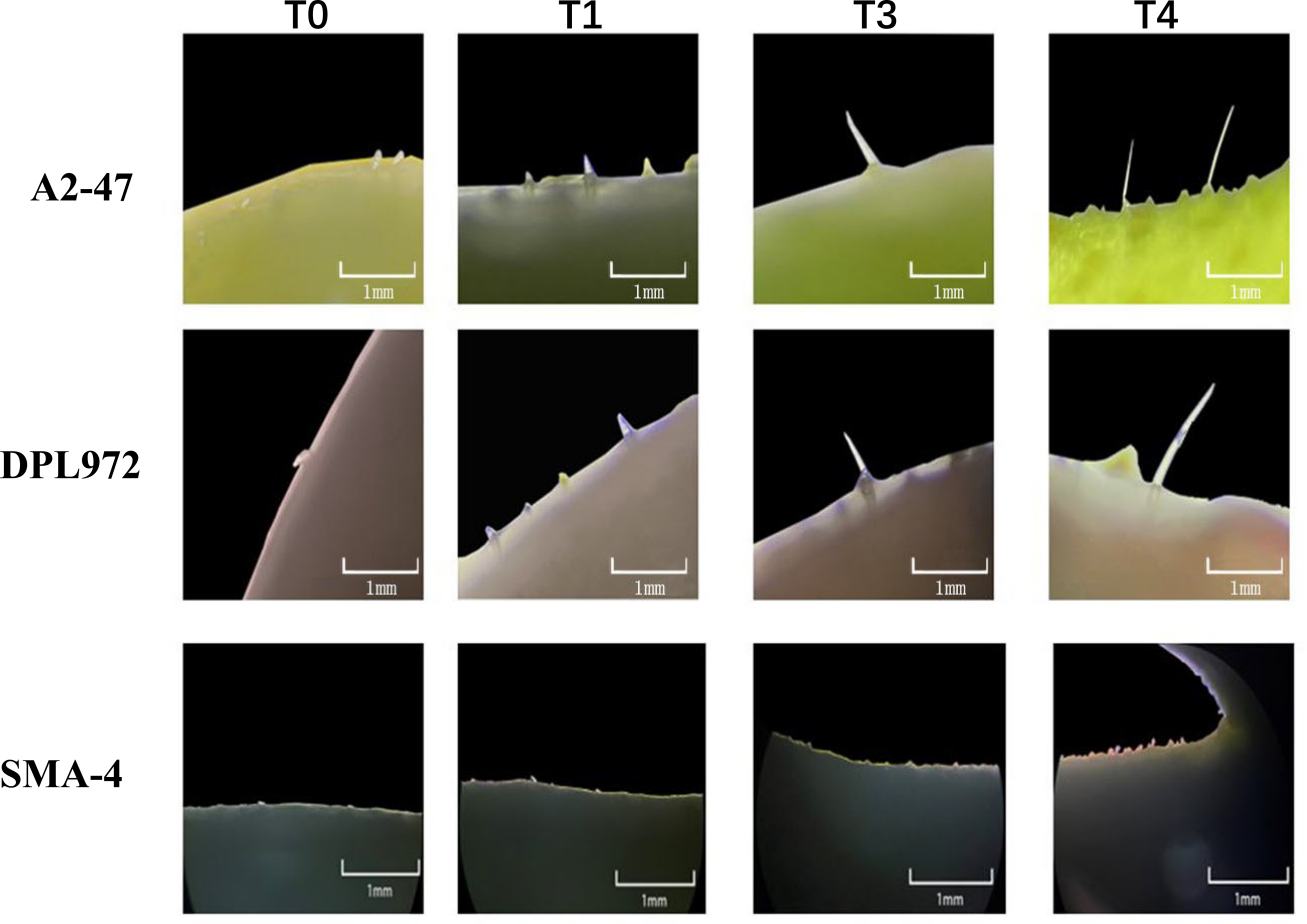
Images of trichome initiation process on hypocotyl of A2-47, DPL972, and SMA-4 under stereomicroscope.

To investigate the initial development of trichomes on the hypocotyl and elucidate the timing of morphological differences in trichomes, seeds were examined under a stereomicroscope following the exposure of the radicle. Observations revealed that 24 hours after radicle exposure, the hypocotyls of all three materials exhibited only round, shield-shaped cell processes at their tips, likely representing glandular hairs. After 60 hours, sharp cell processes were observed in both A2–47 and DPL972, indicating the presence of non-glandular hairs. By 72 hours, the majority of hair cells continued to elongate, and by 104 hours, the lengths of the trichomes stabilized (**Figure 2**). Accordingly, we categorized the time points of 24, 60, 72, and 104 hours post-radicle exposure into four developmental stages: pre-initiation, initiation, elongation, and fixed length, respectively.

**Figure 3:**
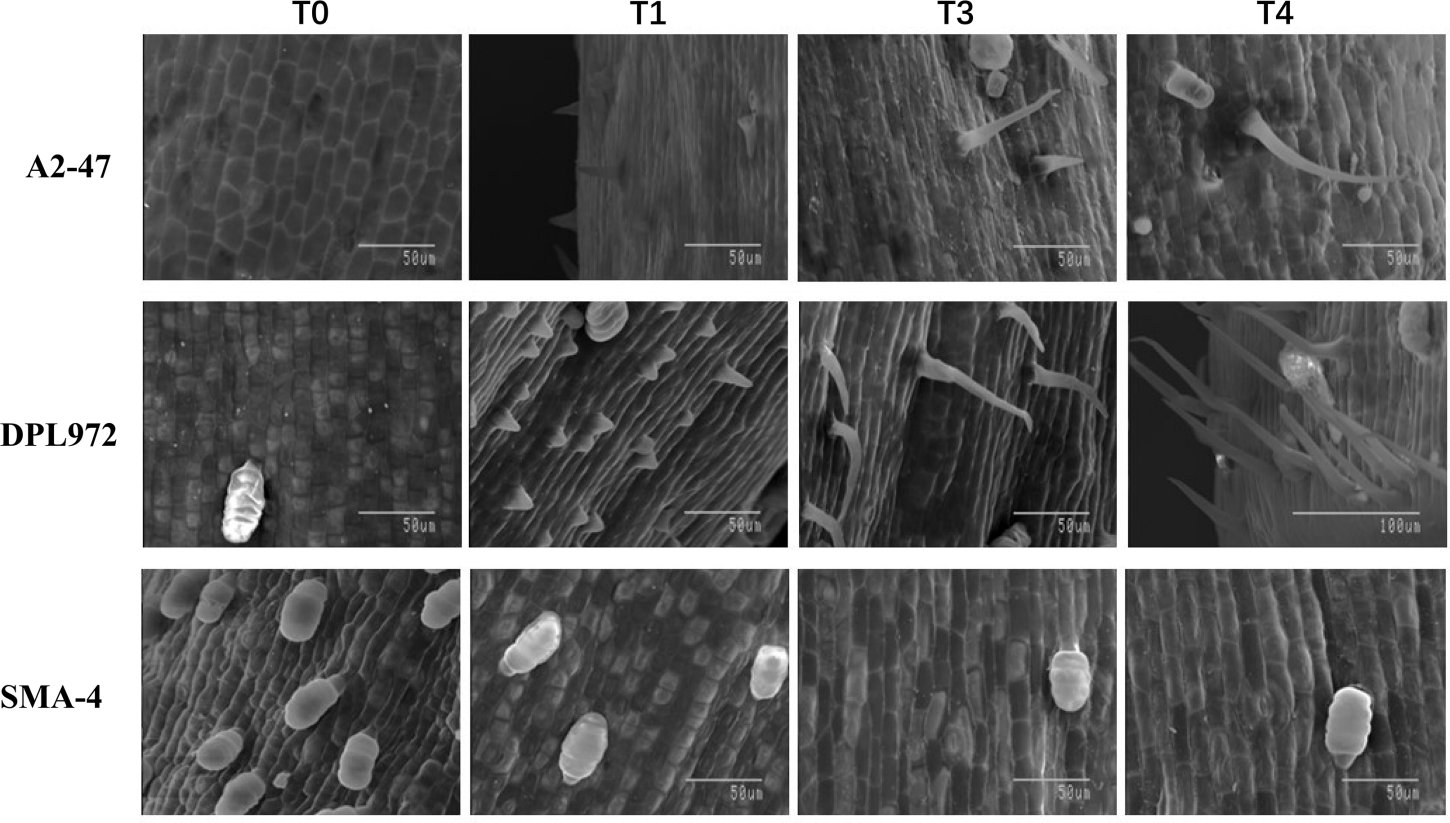
Images of trichome initiation process on hypocotyl of A2-47, DPL972 and SMA-4 under scanning electron microscopy.

At four developmental stages, the epidermis of the hypocotyls was removed for scanning electron microscopy observation, and the results were consistent with those observed under a stereomicroscope: at 24 hours, no protrusions appeared in the epidermal cells; at 60 hours, protrusions began to emerge; and the cells gradually elongated until they reached their final length between 72 and 104 hours (**Figure 3**). Furthermore, clustered hairs that differed from the branched clustered hairs of *Arabidopsis* were observed on the hypocotyl surfaces of DPL972 and A2-47, with the roots of these clusters closely attached to each other (**Supplementary Figure S2**). In this context, we speculate that the developmental pattern of these hairs may involve the simultaneous elongation of two or more closely adjacent epidermal hair cells, as well as a single cell producing two or more cells at the base through cell proliferation (such as asymmetric division or cell rearrangement following lateral division), which then further develop and arrange to form non-single root hairs.

### 3.2 DEGs analysis of epidermal hair cells in hypocotyls of *Gossypium arboreum* at different developmental stages based on PCA analysis

In this study, we constructed 36 cDNA libraries to sequence the epidermal hairs of the hypocotyls of three Asian cotton cultivars across four developmental stages. We obtained a total of 447.6 G of clean sequencing data, with an average data size of approximately 12.4 G per sample. The quality score Q20 exceeded 95.84%, while the Q30 quality score surpassed 90.00% (**Supplementary Tables S1**, **S3**), indicating a high-quality data output. Principal component analysis (PCA) results demonstrated that the biological replicate samples of the three Asian cotton cultivars exhibited strong clustering consistency, suggesting that the differences between developmental stages were more pronounced than those between the varieties. All samples were categorized into four main clusters, corresponding to the preinitial stage (24 h), initial stage (60 h), elongation stage (72 h), and fixed length stage (104 h) of trichome development, with samples at the same developmental stage clustering closely together (**Supplementary Figure S3**).

**Figure 4:**
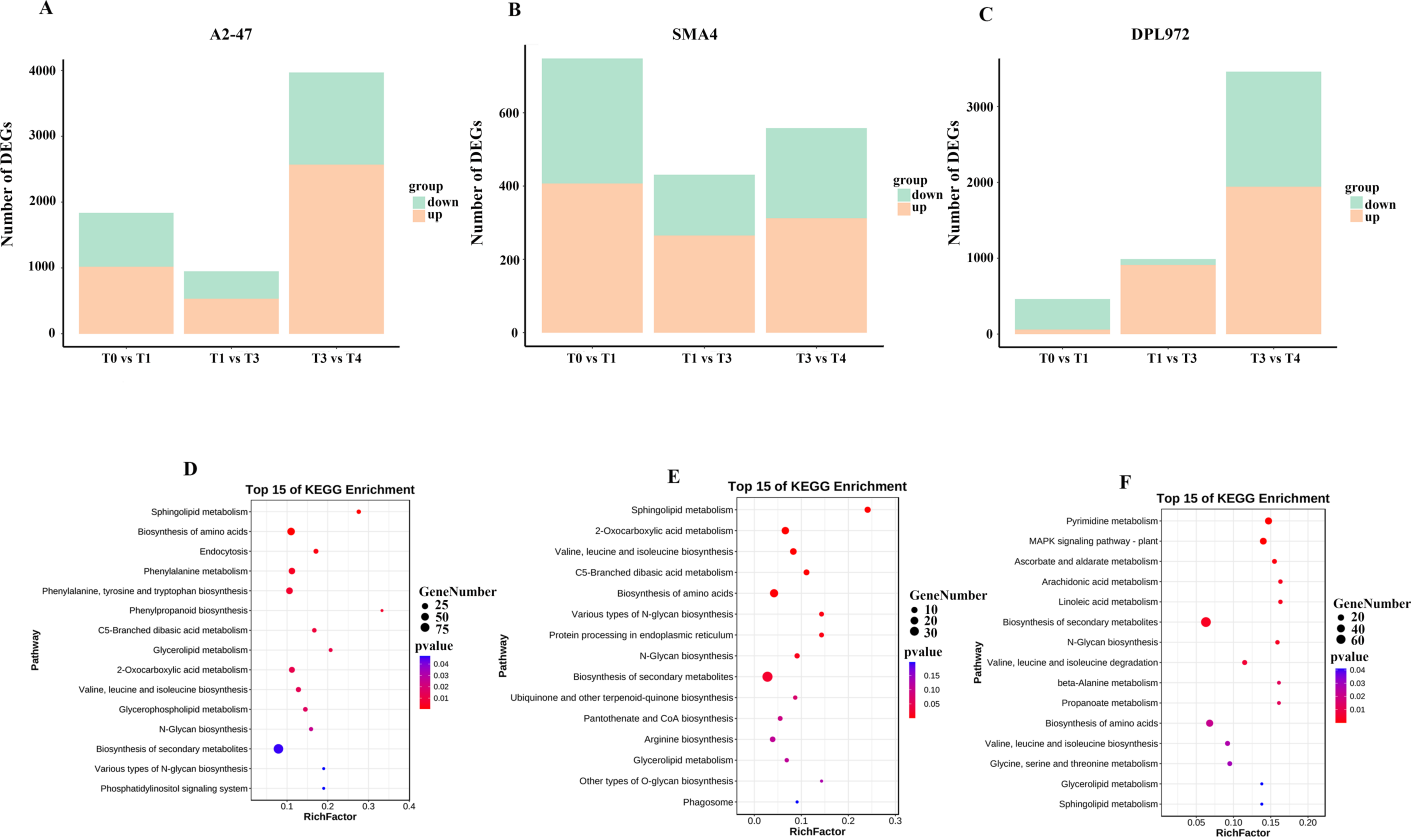
Statistical chart of differential expressed genes. **(A)** A2-47 DEGs statistical chart. **(B)** SMA-4 DEGs statistical chart. **(C)** DPL972 DEGs statistical chart green indicates the number of down regulated genes, orange indicates the number of up-regulated genes. **(D)** A2-47 DEGs KEGG enrichment analysis diagram. **(E)** SMA-4 differential gene KEGG enrichment analysis diagram. **(F)** DPL972 differential gene KEGG enrichment analysis diagram.

Cotton trichome development is a dynamic and complex process regulated by numerous networks and a large array of genes. To identify the differentially expressed genes (DEGs) involved in the initiation and development of Asian cotton hypocotyls, we compared and analyzed two adjacent developmental stages across three materials. In A2-47, a total of 1,837 DEGs were identified by comparing stages T0 and T1, with 1,020 DEGs up-regulated in T1, potentially facilitating the transformation of epidermal cells into trichomes, while 817 DEGs were down-regulated, likely inhibiting changes in epidermal cell morphology. As development progressed, we observed 953 DEGs between T1 and T3, and as many as 3,969 DEGs between T3 and T4, with these DEGs exhibiting varying upward and downward trends across T3 and T4 (**Figure 4A**; **Supplementary Table S4**). To further investigate the biological functions of these DEGs with different regulatory patterns, we conducted a KEGG enrichment analysis, which revealed that all identified DEGs were primarily associated with phenylpropanoid biosynthesis, phenylalanine metabolism, and the biosynthetic pathways of secondary metabolites (**Figure 4D**).

Similarly, in SMA-4, 748 DEGs were obtained in T0 and T1, 431 in T1 and T3, and 558 in T3 and T4 (**Figure 4B**; **Supplementary Table S5**). KEGG enrichment analysis showed that these genes were significantly enriched in the biosynthesis of valine, leucine and isoleucine, biosynthesis of secondary metabolites, and sphingolipid metabolism pathway (**Figure 4E**).

For DPL972, a total of 462 DEGs were identified between T0 and T1, 988 DEGs between T1 and T3, and 3,459 DEGs between T3 and T4 (**Figure 4C**; **Supplementary Table S6**). KEGG enrichment analysis revealed that these genes were significantly associated with pathways involved in secondary metabolite biosynthesis, the MAPK signaling pathway (in plants), and pyrimidine metabolism (**Figure 4F**).

In conjunction with the aforementioned results, the number of DEGs at the T0-T1 and T3-T4 stages was found to be greater than that observed in the other stages, indicating that these two critical periods may play a significant role in regulating the initiation and development of hypocotyl trichomes.

### 3.3 Screening candidate genes for trichome initiation based on maSigpro time series analysis

#### 3.3.1 Time series analysis of SMA-4 and A2–47 to screen candidate genes for trichome initiation and development:

**Figure 5:**
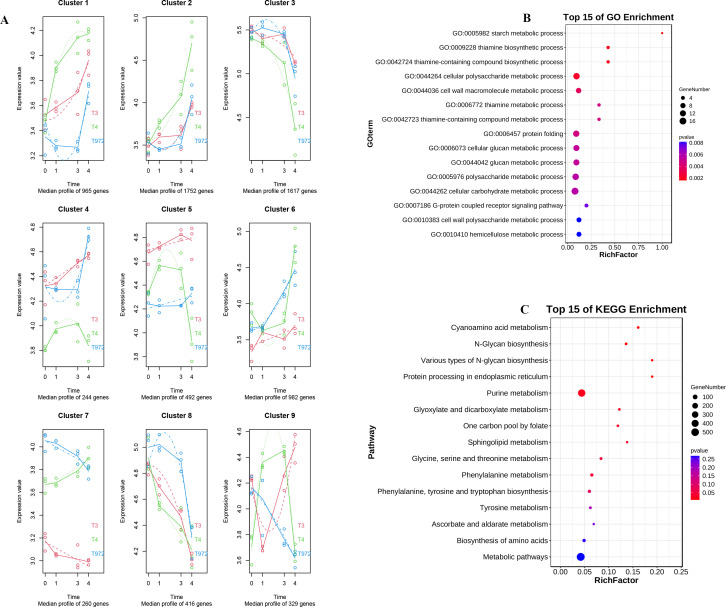
SMA-4 and A2-47 timing analysis. **(A)** Time sequence analysis Multi-segment line graph of differential genes in A2-47 and SMA-4. **(B)** GO enrichment analysis of differential genes in C2 and C3 modules. **(C)** KEGG enrichment analysis of C2 and C3 module differential genes.

In order to understand the expression patterns of DEGs at different stages of puberty initiation and development. SMA-4 and A2–47 were further explored through maSigpro time series analysis (**Supplementary Figure S4**). According to the difference of expression trend, these genes were divided into nine modules, cluster1-cluster9. The genes in cluster6, cluster7 and cluster9 modules (**Supplementary Table S7**) have opposite expression trends before and during the trichome initiation of the two materials (**Figure 5A**). In order to clarify the biological functions and pathways of these three module genes, we performed GO enrichment and KEGG enrichment analysis. GO enrichment analysis results showed that these genes were closely related to starch metabolism, cell wall Macromolecule Metabolism, cell wall polysaccharide metabolism and hemicellulose metabolism. These processes had significant effects on plant cell division, differentiation and elongation, as well as the construction and maintenance of cell wall structure. At the same time, KEGG enrichment analysis also showed that these differential genes were significantly enriched in the biosynthesis of phenylalanine, tyrosine and tryptophan, phenylalanine metabolism, glycine, serine and threonine metabolism and other pathways, which played an important role in plant growth and development, metabolism and stress response pathways (**Figures 5B, C**).

#### 3.3.2 Screening candidate genes for trichome initiation by DPL972 and SMA-4 time series analysis

**Figure 6:**
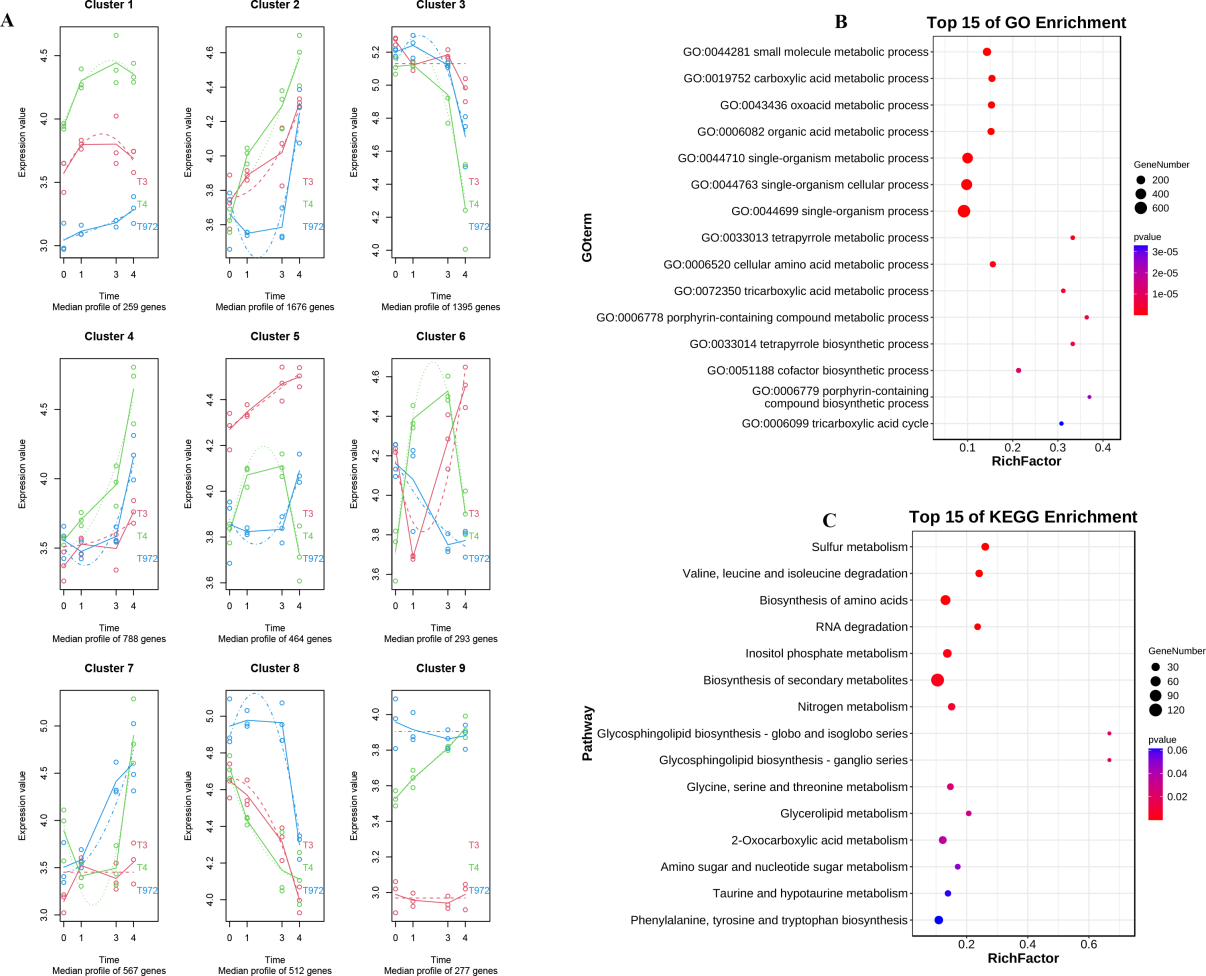
DPL972 and SMA-4 timing analysis. **(A)** Time sequence analysis Multi-segment line graph of differential genes in DPL972 and SMA-4. **(B)** GO enrichment analysis of differential genes. **(C)** KEGG enrichment analysis of differential gene.

MaSigpro time series analysis is also performed on DPL972 and SMA-4 (**Supplementary Figure S5**). The results showed that the genes in cluster3, cluster4 and cluster5 modules (**Supplementary Table S8**) had opposite expression trends before and during the trichome initiation of the two materials (**Figure 6A**). GO enrichment and KEGG enrichment analysis were performed. The results of GO enrichment analysis showed that the DEGs were related to biological processes such as oxygenic acid metabolism, carboxylic acid metabolism and small molecule metabolism. KEGG enrichment analysis is closely related to biosynthesis of secondary metabolites, biosynthesis of amino acids, and degradation pathways of valine, leucine, and isoleucine (**Figures 6B, C**).

### 3.4 Analysis of the regulatory network of cotton hairy morphogenesis related genes based on WGCNA

**Figure 7:**
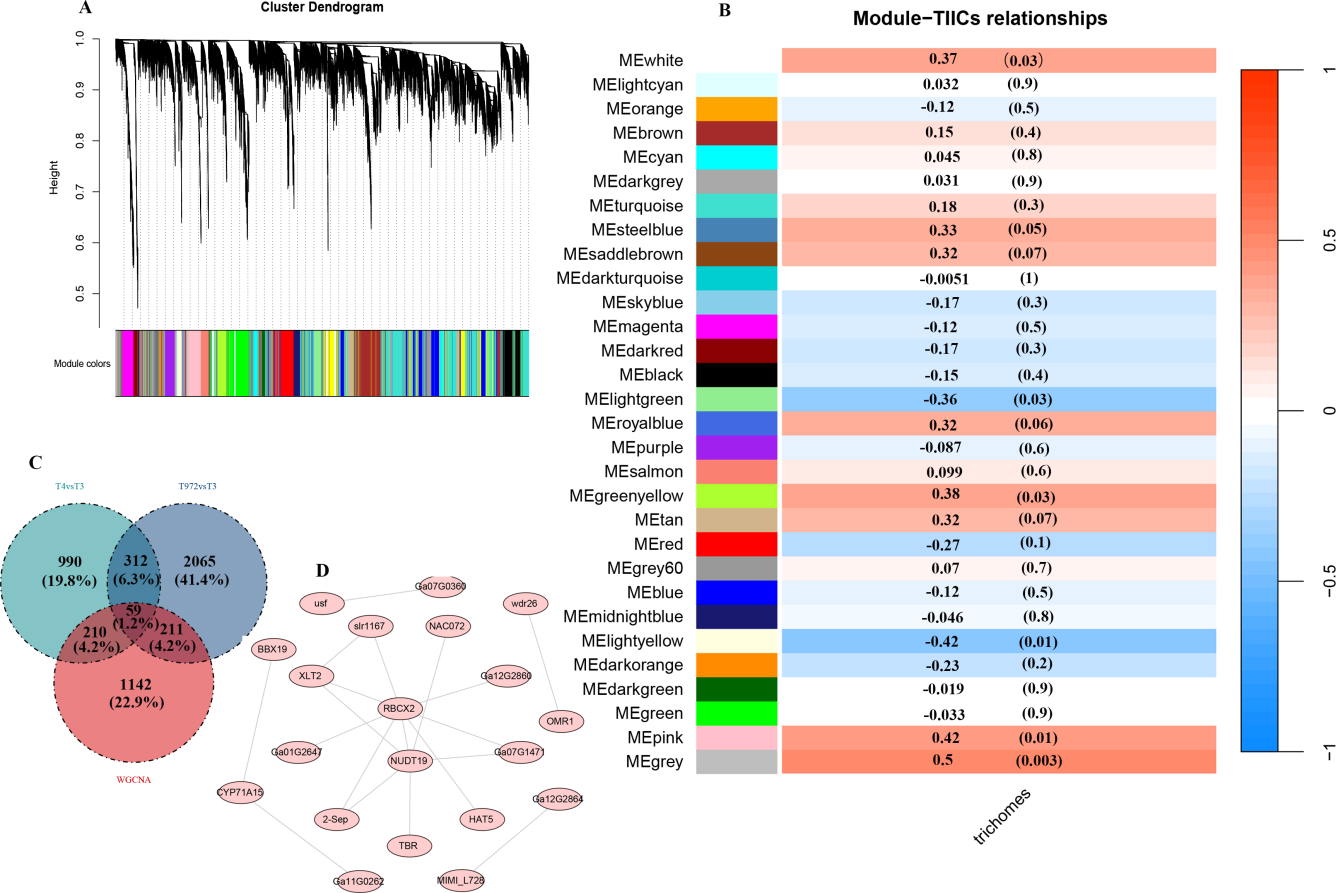
WGCNA plot. **(A)** Hierarchical clustering tree revealing co-expression module identification. **(B)** Correlation of module characterized genes with traits. **(C)** Venn diagram of WGCNA module genes with maSigpro temporal differential genes. **(D)** Co-expression network of candidate genes.

The transcriptome data were analyzed globally to explore the phenotypic association between DEGs and trichome morphogenesis and transformation. The transcriptome data were processed by WGCNA. All genes were divided into 30 modules, and each module was represented by a separate branch (**Figure 7A**). These modules are composed of genes that exhibit similar expression patterns, reflecting their close relationship in function or regulation. Compared with other modules, MEpink module was positively correlated with trichome morphogenesis, while MElight yellow module was negatively correlated with trichome morphogenesis (cox = 0.42, r = -0.42, P value < 0.05) (**Figure 7D**). MEpink module contains *GaHD1 (Ga06G1792), GaRDL1 (Ga05G0755), GaEXPA1 (Ga10G1805)* and other genes closely related to the initiation and development of epidermal hair (**Supplementary Table S9**) (Xu et al., 2013).

To further investigate the functional characteristics of these modules, we conducted KEGG enrichment analysis on the genes associated with the MEpink and MElightyellow modules. The results indicated that the genes in the MEpink module are primarily involved in the biosynthesis of thiometabolism, as well as the metabolism of valine, leucine, and isoleucine, along with the C5-branched-chain dicarboxylic acid metabolic pathway (**Supplementary Figure S6A**). In contrast, the genes in the MElightyellow module exhibit significant enrichment in pathways related to the degradation of valine, leucine, and isoleucine, the phosphatidylinositol signaling pathway, RNA polymerase activity, and carbon metabolism (**Supplementary Figure S6B**).

To identify candidate genes directly associated with the initiation and development of puberty, we analyzed the intersection of differentially expressed genes (DEGs) and genes from weighted gene co-expression network analysis (WGCNA) modules, ultimately identifying 59 intersecting genes (**Figure 7C**). These genes are considered potential candidates for involvement in trichome initiation and development. We constructed a regulatory network for these 59 genes using Cytoscape software and found that only 20 of these genes may have regulatory interactions. Notably, the proteins encoded by Ga13G2472 (NUDT19) and Ga05G0743 (RBCX2) are more likely to interact with other proteins (**Figure 7D**), suggesting their significant roles within the regulatory network.

### 3.5 Machine learning accurately screens key genes for hypocotyl epidermal hair development

To accurately identify the key genes closely associated with the initiation and development of puberty, we utilized a combination of three machine learning methodologies SVM-RFE analysis, Boruta’s algorithm, and LASSO regression curve analysis based on the 59 candidate genes previously identified through weighted gene co-expression network analysis (WGCNA). The integration of these three approaches enhances the stability and accuracy of our feature selection process, thereby constructing a robust predictive model.

**Figure 8:**
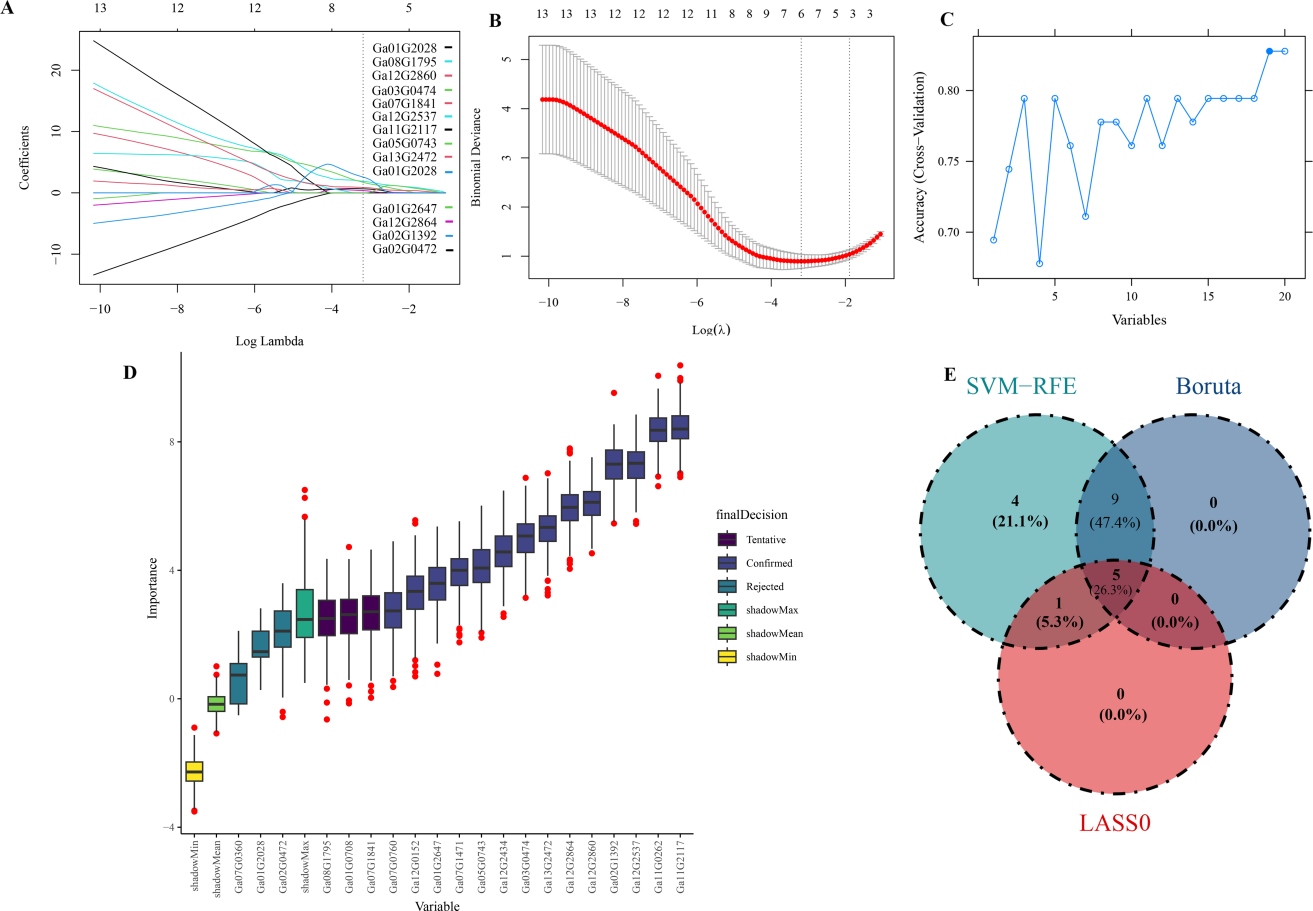
LASSO regression curves. **(A)** LASSO regression roadmap: the abscissa is the logarithm of Lambdas, and the ordinate is the variable coefficient. With the increase of Lambdas, the variable coefficient approaches zero. When the optimal lambda is reached, variables with a coefficient equal to 0 are eliminated. **(B)** LASSO cross validation diagram: the abscissa is the logarithm of Lambdas, and the ordinate model error. The best lambda value is at the lowest point of the red curve. **(C)** SVM-RFE analysis chart: the abscissa represents the performance index of the model, the ordinate represents the number of genes. **(D)** Boruta analysis chart: the abscissa represents the name of variables, and the ordinate represents the importance of features. **(E)** Three machine learning Venn charts.

In the LASSO regression curve analysis, when the optimal λ value (0.04107188) is reached, six genes are not punished to 0, which indicates that these six genes are more important in the process of pubescence growth and development than other genes (**Figures 8A, B**). In SVM-RFE analysis, when the model reached the highest correlation for the first time, 19 genes passed the algorithm verification (**Figure 8C**), which showed that these genes had a high contribution in predicting the initial development of trichome. At the same time, Boruta’s algorithm was also used to analyze. The results showed that 13 genes passed the model validation (**Figure 8D**). The intersection results are shown in three machine learning models, *Ga02G1392(TBR)*, *Ga03G0474(OMR1)*, *Ga12G2860(ACO1)*, *Ga11G2117(BBX19) and Ga12G2864(CUE)* both can pass the verification (**Figure 8E**). These results indicate that these five genes play an important role in the initial development of trichome.

### 3.6 Expression trend analysis of DEGs during the initial development of epidermal hair

**Figure 9:**
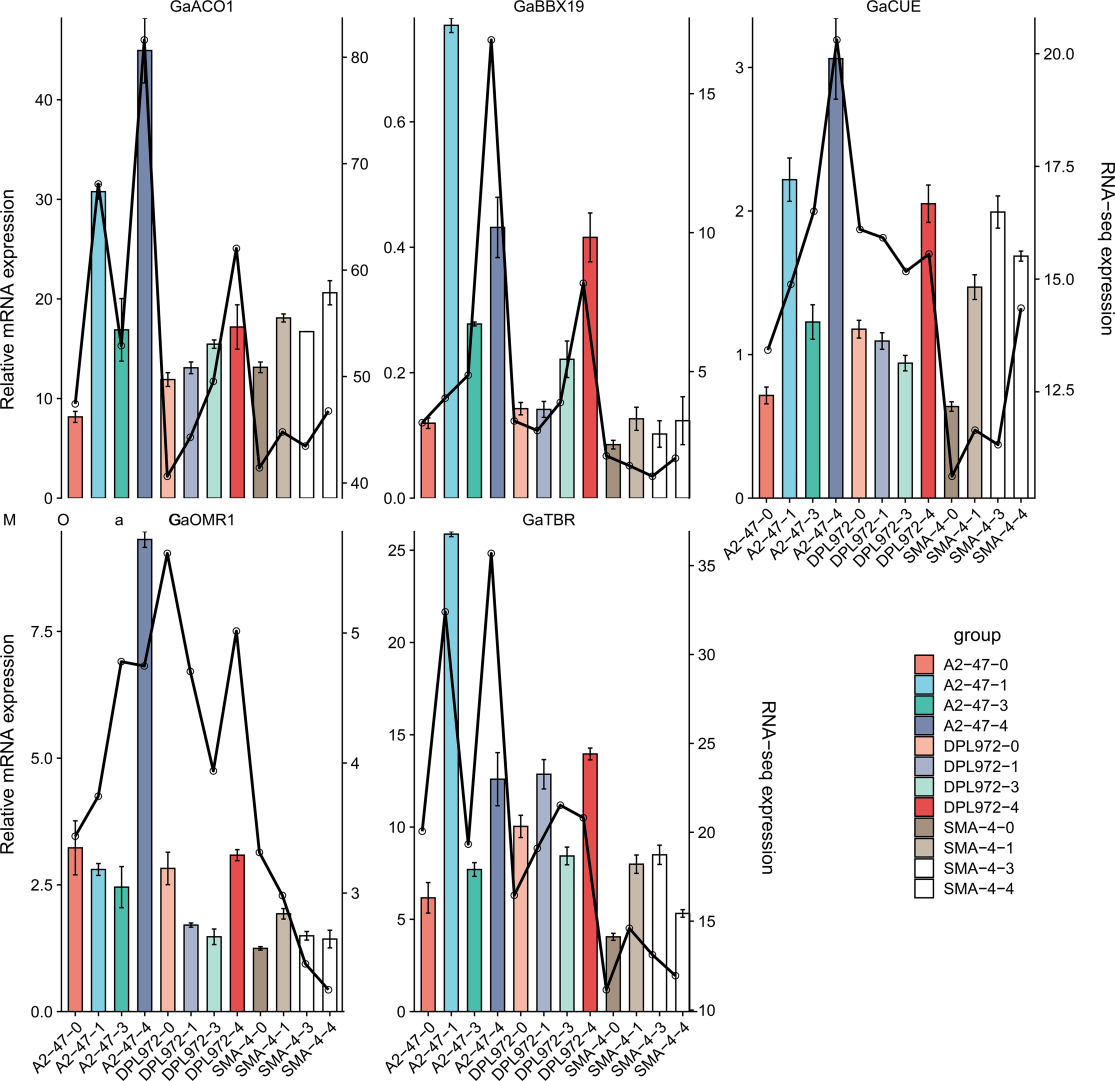
Trend of differential gene expression level. The abscissa is the initial development stage of the epidermis, and the ordinate is the relative expression. The histogram shows the relative expression of RT qPCR; The line graph represents the relative expression in transcriptome data.

In order to further verify the reliability and accuracy of RNA seq data, we used real-time fluorescent quantitative PCR to analyze the expression levels of five screened genes *Ga02G1392 (TBR), Ga03G0474 (OMR1), Ga12G2860 (ACO1), Ga11G2117 (BBX19)*, and *Ga12G2864 (CUE)*. The RT qPCR results were compared with the transcriptome analysis data. The results showed that the relative expression level obtained by RT qPCR analysis was consistent with the TPM value calculated by RNA seq (**Figure 9**), indicating that the bioinformatics analysis method used in RNA seq data processing was correct.

At the same time, the expression level trend of five genes during the initial development of hypocotyl was analyzed (**Figure 9**). The results showed that the expressions of *GaTBR* and *GaBBX19* in A2–47 were up-regulated at the initial stage, and the expressions of *GaACO1* and *GaCUE* gradually increased at the secondary cell wall thickening stage, while the expressions of *GaOMR1* and *GaTBR* increased again at the mature stage, indicating that the expression trend of the five genes in normal gene expression materials showed obvious stage specificity, which was highly consistent with the four stages of development. The expression levels of *GaTBR, GaBBX19, GaOMR1* and *GaACO1* genes in SMA-4 were significantly lower than those in A2-47, while *GaTBR* was highly expressed in DPL972, while *GaCUE* was very low. These results suggest that the disorder of gene expression sequence (inhibition or continuous activation) in the mutant may be the reason for the difference of trichome phenotype.

## 4 Discussion

### 4.1 Cotton hypocotyl epidermal trichome: a good system for studying the fate of epidermal cells

Analyzing the molecular mechanisms that drive the differentiation and development of plant epidermal cells remains a central focus of studies in plant organogenesis and morphological diversity. In this research domain, establishing an optimal experimental system is crucial for ensuring successful outcomes. Plant epidermal hairs, commonly referred to as trichomes, have emerged as a paradigmatic model for investigating plant cell differentiation—specifically, the determination of cell fate and the regulation of polarity, owing to their ease of visualization and single-cell nature (Hulskamp, 2004). Trichomes of Arabidopsis thaliana have been extensively utilized as model systems, capitalizing on their genomic characteristics and the organism’s status as a model plant (Han et al., 2022), thereby elucidating the molecular mechanisms underlying trichome development.

Although a single-cell development model for cotton fibers has been established (Qin et al., 2022), the sampling of ovule epidermal cells in cotton is feasible only during specific growth stages, namely pre- and post-flowering. The small size of these cells, as well as their encapsulation within the ovary, presents significant challenges for sampling and observation. Both cotton fiber and stem fuzz are composed of single cells, and the cytological processes through which ovule cells extend to form cotton fibers display similarities to the formation of stem fuzz. Research has revealed notable parallels in their molecular regulatory mechanisms; thus, exploring the molecular pathways involved in the development of stem and leaf hairs is crucial for advancing our understanding of the mechanisms underlying fiber development.

To date, research on cotton stem trichomes has primarily focused on measuring trichome length and classifying trichome types in their mature states (Yuan et al., 2021). The findings from these studies may be somewhat outdated and could overlook the underlying molecular mechanisms governing epidermal cell differentiation. This study reveals that during the germination of Asian cotton seeds, specific epidermal cells in the hypocotyl initiate protrusion and develop into trichomes, which provide an accessible subject for observation and sampling. Consequently, we present a straightforward and efficient system for investigating plant epidermal cell differentiation and development, offering a model for exploring the fate of essential epidermal cells in crops. Utilizing this system, we compared dynamic changes in epidermal hair phenotypes between the wild-type A2–47 and the mutants SMA-4 (GaHD1 mutation) and DPL972 (which exhibits high expression of GaGIR1), identifying four distinct stages in the differentiation and development of epidermal cells into trichomes. Each stage was characterized by unique features: the preinitial stage (T0) exhibited condensed basal chromatin without nuclear enlargement; the initial stage (T1) displayed polar protrusions at the cell apex and nuclear translocation to the sub-apex; while the elongation stage (T3) and the fixed length stage (T4) were distinguished by ongoing cell elongation and specific nuclear localization.

Furthermore, this study first observed that the formation of clustered, non-single epidermal trichomes involves two pathways: proliferation and rearrangement of epidermal hair cells, alongside simultaneous development and growth of adjacent cells. This offers a novel research perspective on plant cell division and regulation of trichome polarity, providing crucial morphological evidence to further unravel the molecular mechanisms underlying cotton fuzz development.

### 4.2 Innovative application of key gene screening technology for epidermal hair initiation and development

The innovative application of key gene screening technology for epidermal hair initiation and development in this study has achieved a dual breakthrough in the methodology for identifying such genes.

#### 4.2.1 Dynamic capture of DEGs at multiple stages

Utilizing the precise time series system (T0-T4) of Asian cotton epidermal hair development, this study employed the MaSigPro two-step regression method to effectively analyze dynamic DEGs between the wild-type A2–47 and mutants (SMA-4, DPL972). Compared to traditional differential analysis, this approach significantly enhances the recognition sensitivity of developmental sequence-specific genes, as previously reported (Nueda et al., 2014). This analysis method is widely used in medicine to identify the gene expression with time changes in different cell types. The common response of immune cells to virus infection at different times (O'Leary and Zheng, 2024). In this study, 1572 and 2648 DEGs were identified between SMA-4 and A2–47 and between DPL972 and A2-47, which improved the capture efficiency of developmental stage specific genes.

#### 4.2.2 Multimodal machine learning fusion screening

In pepper (*Capsicum annuum L.*), WGCNA was used to screen important modules related to trichome formation, and six differential genes related to trichome development in Pepper epidermis (Shen et al., 2024) were identified. To mitigate the biases inherent in a single algorithm, this study innovatively integrates WGCNA with three machine learning algorithms: LASSO, SVM-RFE and Boruta feature selection analysis. Machine learning can integrate genome, transcriptome, epigenome and other multi omics data, find cross level marker combinations, and improve predictability. Machine learning can mine potential markers (such as non coding RNA and microbiome characteristics) ignored by traditional methods, and reveal their biological mechanisms (GuyonIsabelle and ElisseeffAndré, 2003; Kursa and Rudnicki, 2010; Xiaokang, 2020). However, in bioinformatics, data sets are often complex and the number of samples is limited. A single model is prone to fitting problems, that is, it performs well on the training set, but has poor generalization ability on unknown data (Bolón-Canedo et al., 2012, Saeys et al., 2007). In addition, a single machine learning algorithm may be sensitive to some characteristics of the data or noise, resulting in different results of the screening basis (Statnikov et al., 2008; Subramanian et al., 2005) given by different algorithms. Therefore, by selecting the intersection of different machine learning algorithms and combining the advantages of various machine learning algorithms (SVM-RFE, Lasso, Brouta), we can reduce the possible deviation caused by a single algorithm. A more comprehensive and diversified result is obtained, which helps to reduce noise, avoid over fitting, improve biological credibility, and ensure that the five different genes screened can be consistently verified in multiple algorithms, thus enhancing the reliability of the results (Statnikov et al., 2008; Subramanian et al., 2005).

### 4.3 Functional network analysis of key candidate genes

We conducted an analysis of the roles played by five key genes identified through our screening in the regulatory network governing cotton trichome initiation and development. The formation of epidermal hair papillae is a crucial aspect during the cell wall maturation stage (Esch et al., 2003; Hulskamp et al., 1994; Kawashima et al., 2014). In *Arabidopsis*, the *TBR* protein regulates the deposition of crystalline cellulose in the papillae region, which in turn affects trichome transparency and structural integrity (Sinclair et al., 2020; Suo et al., 2013). Our findings suggest that the acetyltransferase encoded by *GaTBR* may also participate in cotton trichome development through a similar mechanism. Xyloglucan, an essential structural polysaccharide in plant cell walls, forms cross-linking structures with cellulose microfibrils that restrict cell elongation (Smith and Hake, 1992; Vissenberg et al., 2005). Consequently, *GaTBR* may influence cell wall loosening and cell elongation by modulating xyloglucan acetylation levels, thereby affecting the activity of enzymes such as *XTH* (Vissenberg et al., 2000, 2005). Importantly, *GaTBR* exhibits strictly developmental stage-specific expression in wild-type plants, while its expression is disrupted in mutants, indicating potential direct or indirect regulation by the upstream transcription factor *GaHD1*.

Branched-chain amino acids (BCAAs) are essential building blocks for protein synthesis, and their metabolites also function as signaling molecules that regulate cell cycle progression. In *Arabidopsis*, the threonine deaminase/dehydratase (TD) encoded by *OMR1* plays a critical role in coordinating cell proliferation and expansion during root hair development by catalyzing isoleucine biosynthesis (Yu et al., 2013). This study posits that *GaOMR1* may influence cell proliferation and morphogenesis in cotton trichome development through the modulation of isoleucine biosynthesis.

The BBX protein family, identified as core regulators of photomorphogenesis (Gangappa and Botto, 2014), is capable of modulating trichome initiation by integrating various endogenous hormone signaling pathways (Wang et al., 2024; Xin et al., 2025; Xu et al., 2024). In cotton, the DELLA protein GhSLR1, which acts as a negative regulator of gibberellin (GA) signaling, suppresses fiber cell elongation by destabilizing the GhHOX3-GhHD1 complex (Hao et al., 2012). Conversely, *GhTCP14* promotes GA biosynthesis, induces the degradation of GhSLR1 protein, and activates downstream cell wall loosening genes such as *GhRDL1* and *GhEXPA1*, ultimately facilitating fiber elongation (Shan et al., 2014). Building on these findings, we hypothesize that *GaBBX19* in Asiatic cotton (*Gossypium arboreum*) is involved in the hormone-mediated regulation of trichome development. Its responsiveness to photoperiod signals (Wang et al., 2014) may account for the phenotypic variation observed in trichome density under different light conditions.

Ethylene, a well-known inhibitory hormone, displays a negative correlation between the activity of its key biosynthetic enzyme, 1-aminocyclopropane-1-carboxylate oxidase (*ACO*), and cell elongation (Zarembinski and Theologis, 1994). In *Arabidopsis*, mitogen-activated protein kinase 6 (*MPK6*) phosphorylates 1-aminocyclopropane-1-carboxylate synthase (*ACS*) proteins to enhance ethylene biosynthesis, thereby influencing stress-responsive growth (Liu and Zhang, 2004). These findings suggest that *GaACO1* may regulate trichome cell elongation through the activation of MAPK cascades, the downregulation of cell cycle-related genes, and its involvement in the ethylene biosynthesis pathway (Wang et al., 2011).

In summary, this study employed an established model of cotton hypocotyl trichome initiation to gain deeper insights into the complete life cycle of hypocotyl epidermal trichome cells in cotton. By integrating temporal progression analysis (MaSigPro), co-expression networks (WGCNA), and machine learning approaches, we systematically and comprehensively analyzed the differential expression profiles during the initiation and development of cotton trichomes. Our analysis identified five candidate genes associated with cell wall formation and the gibberellin and abscisic acid signaling pathways, which are likely to serve as key regulators of trichome initiation in Asiatic cotton (*Gossypium arboreum*) hypocotyls. This research provides a detailed morphological framework for investigating the molecular mechanisms underlying cotton trichome development and establishes a robust data and theoretical foundation for subsequent functional validation of these candidate genes.

## Data Availability

The original data has been uploaded to NCBI:“PRJNA1282329 : Transcriptomic Exploration Yields Novel Perspectives on the Regulatory Network Underlying Trichome Initiation in Gossypium arboreum Hypocotyl.
