# Supplementary material for: Transcriptomic exploration yields novel perspectives on the regulatory network underlying trichome initiation in Gossypium arboreum hypocotyl
Source: Front Plant Sci. 2025 Jul 2;16:1604186. doi: 10.3389/fpls.2025.1604186 (PMC12263914; doi:10.3389/fpls.2025.1604186)
Supplement: Supplementary file 1 [file DataSheet1.docx]

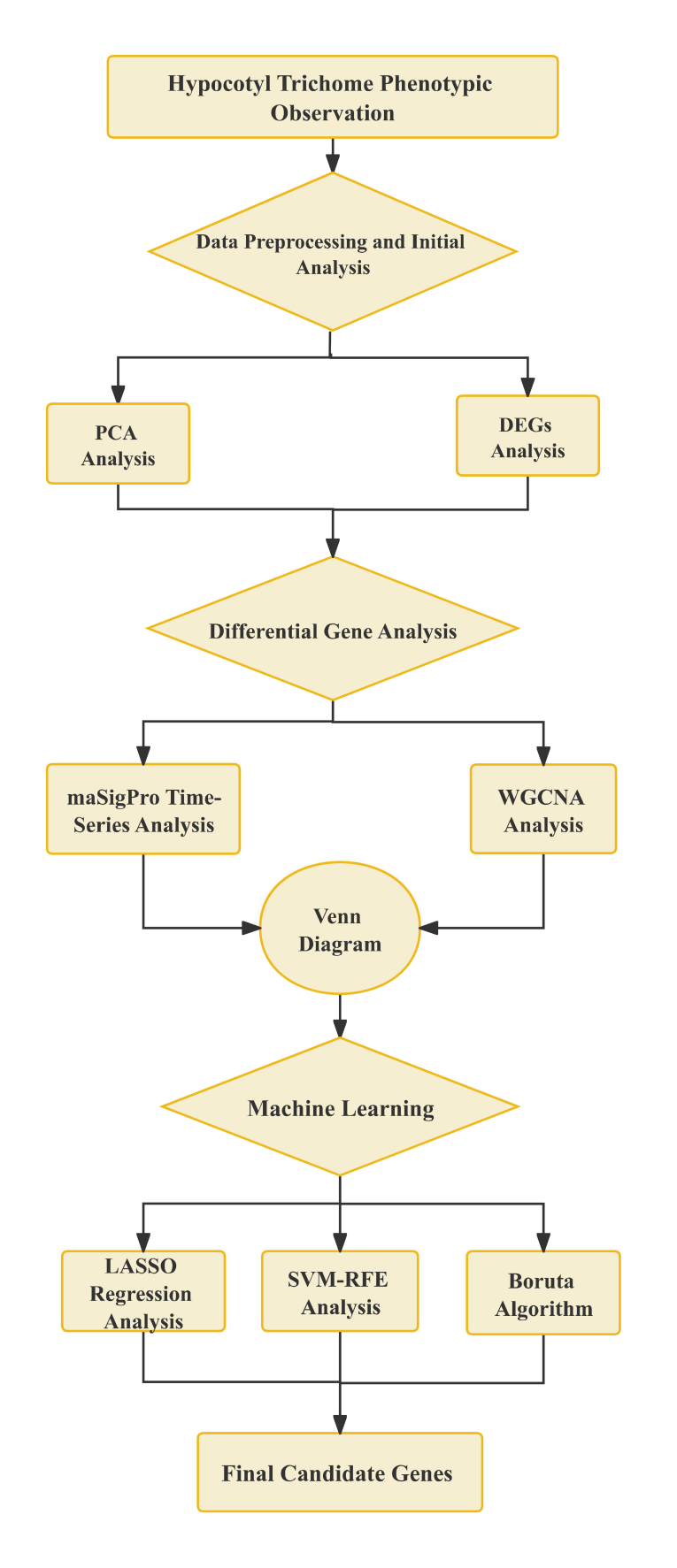


**Fig S1. Technical process**


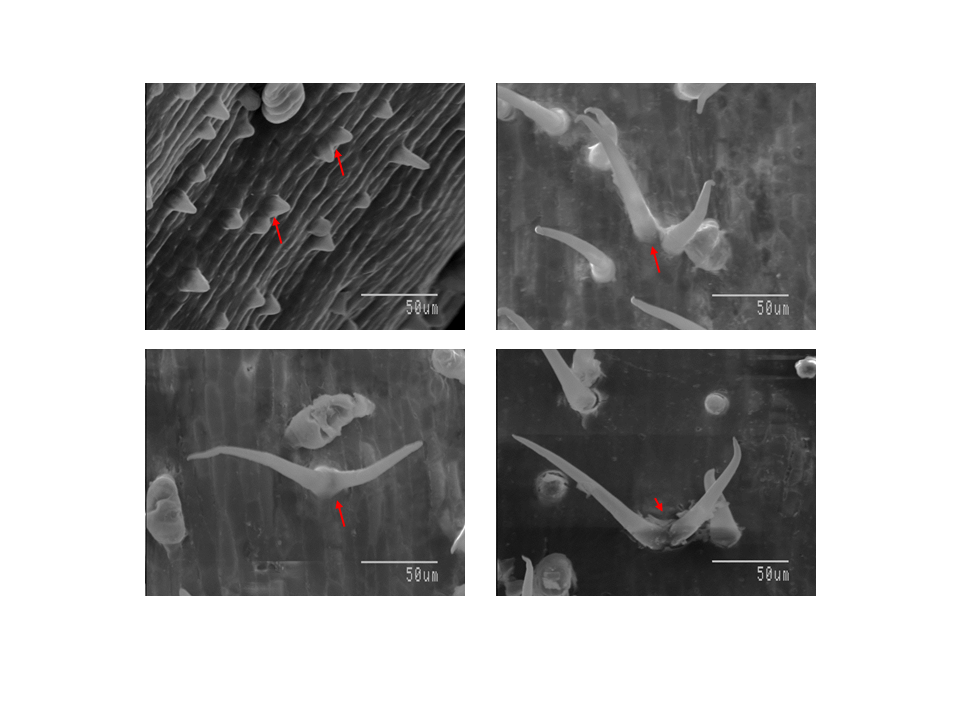


**Fig S2. Scanning electron microscope scale of hypocotyl tufted fluffy cells: 50μm**

**
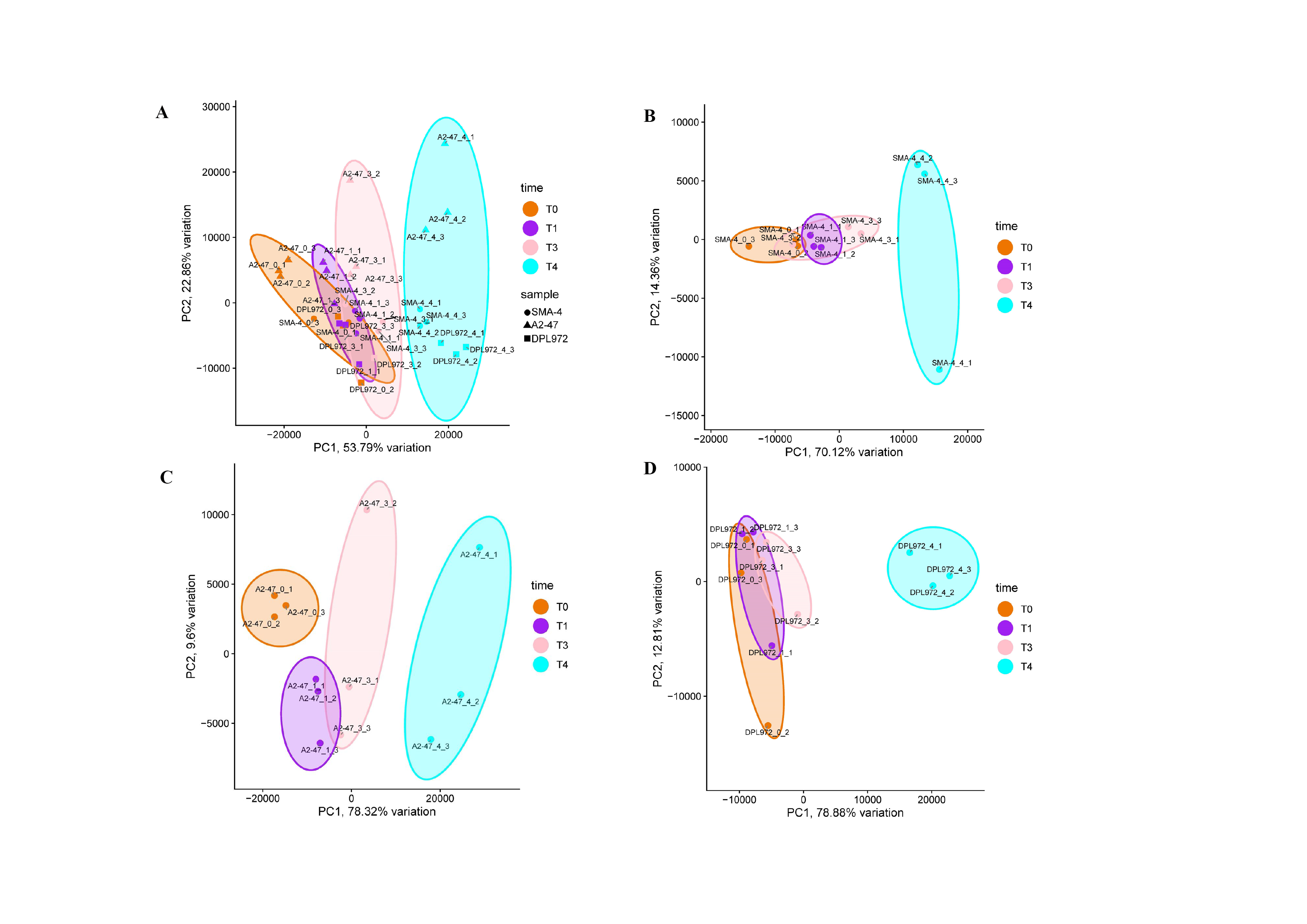
**

**Fig S3. Principal Component Analysis of SMA-4, A2-47 and DPL972 Samples**

**(A)** Principal component analysis (PCA) of SMA-4, A2-47 and DPL972 samples. **(B)** Principal component analysis (PCA) of SMA-4 samples. **(C)** Principal component analysis (PCA) of A2-47 samples. **(D)** By principal component analysis (PCA) of DPL972 samples, T0, T1, T3 and T4 represented 24 h, 62 h, 74 h and 106 h, respectively.


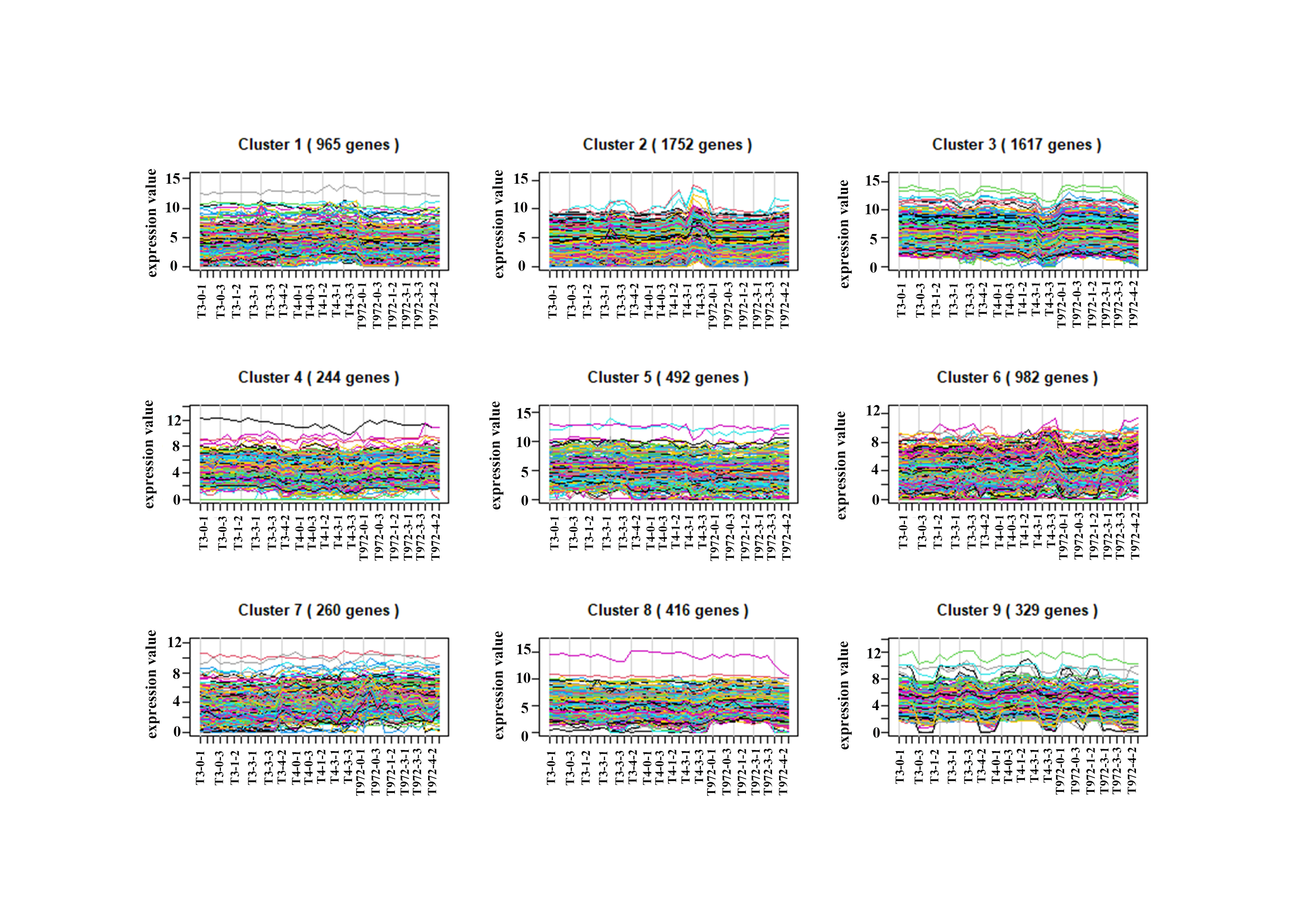


**Fig S4. A2-47 and SMA-4 maSigpro time series analysis diagram**

T3 represents: SMA-4 T4: represents A2-47 0, 1, 3, 4 represent 24h, 60h, 72h, 104h respective Each line represents the expression trend of a gene at different times.


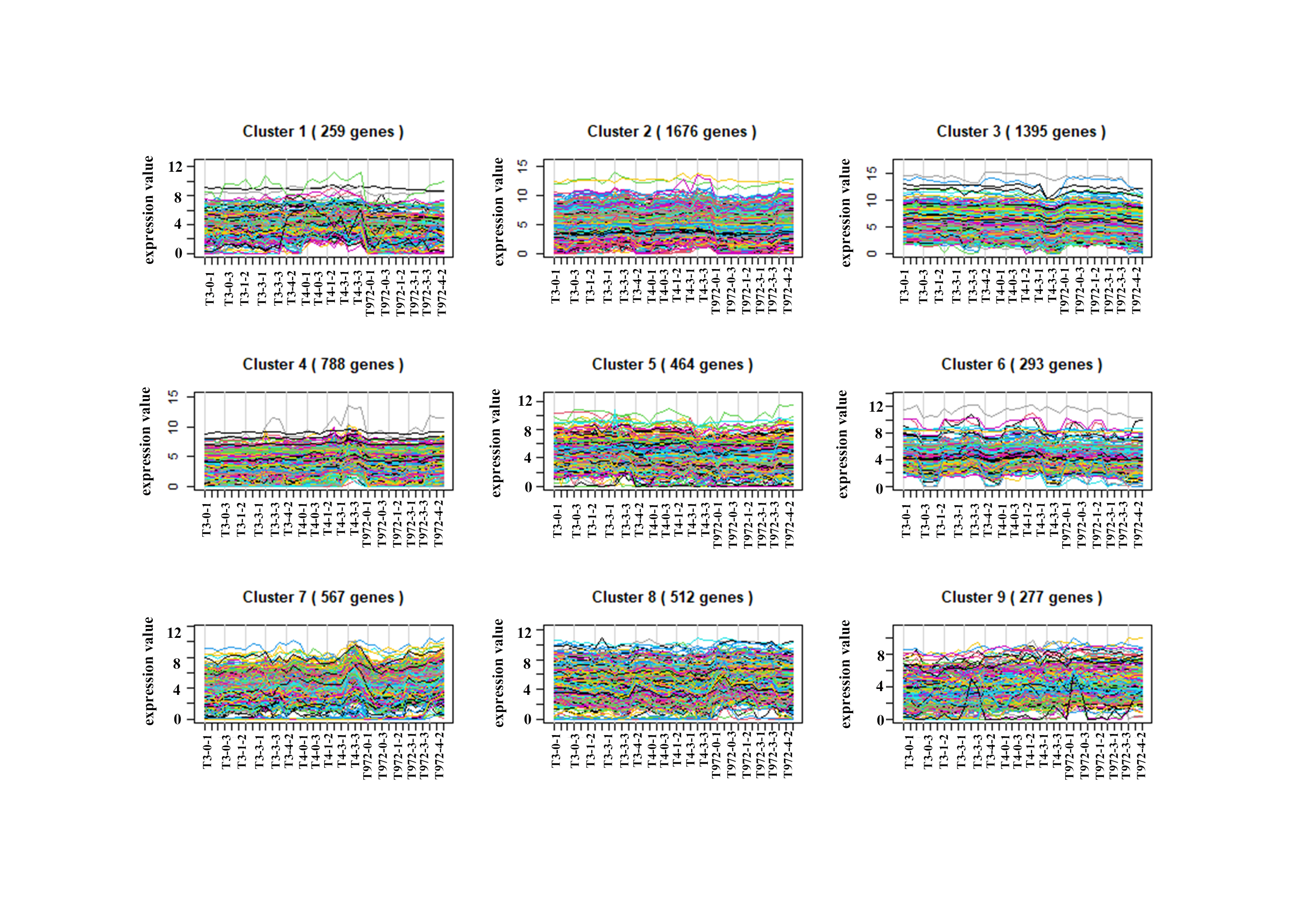


**Fig S5. DPL972 and SMA-4 maSigpro timing analysis diagram**

T3 represents: SMA-4,T972: represents DPL972 0, 1, 3, 4 represent 24h, 60h, 72h, 104h Each line represents the expression trend of a gene at different times.

**
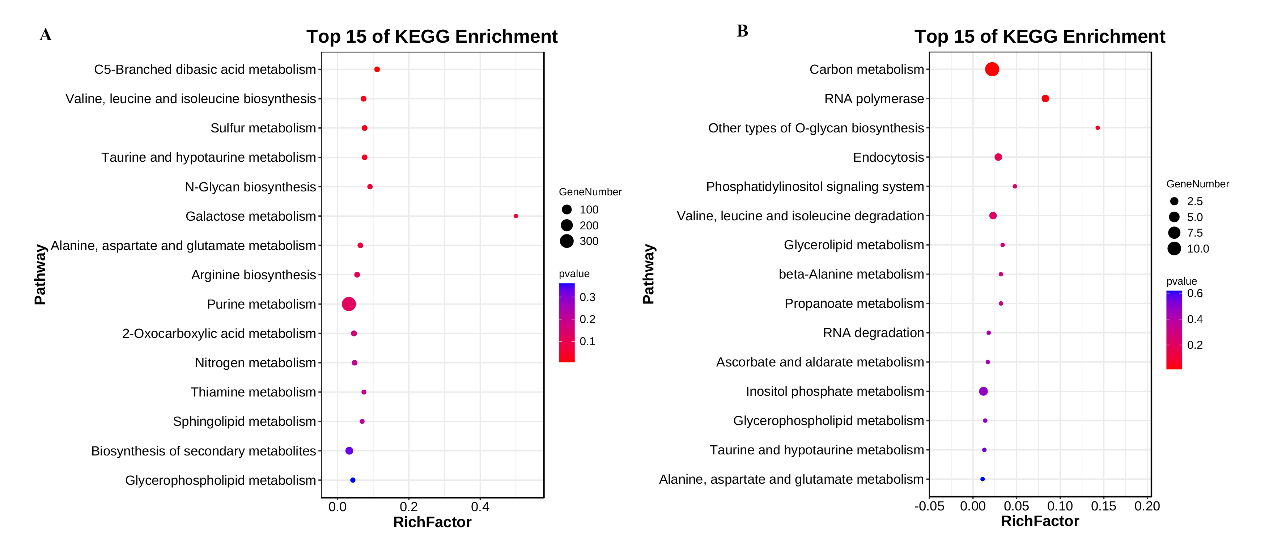
**

**Fig S6. KEGG enrichment analysis of genes in MEpink and melightyyellow modules**

(A) MEpink KEGG enrichment analysis (B) Melightyellow module KEGG enrichment analysis
